# Care Pathways After Acute Myocardial Infarction: A Gender-Based Perspective

**DOI:** 10.3390/jcm15072592

**Published:** 2026-03-28

**Authors:** Irene López-Ferreruela, Lina Maldonado, Sara Malo, María José Rabanaque, Isabel Aguilar-Palacio

**Affiliations:** 1 Epila Health Centre, Primary Care, Servicio Aragonés de Salud (SALUD), 50290 Epila, Zaragoza, Spain; 2 Grupo de Investigación en Servicios Sanitarios de Aragón (GRISSA), Aragon Institute for Health Research (IIS Aragón), 50009 Zaragoza, Spain; lmguaje@unizar.es (L.M.); smalo@unizar.es (S.M.); rabanake@unizar.es (M.J.R.); iaguilar@unizar.es (I.A.-P.); 3 Department of Applied Economy, Universidad de Zaragoza, 50001 Zaragoza, Spain; 4 Research Network on Chronicity, Primary Care and Health Promotion (RICAPPS), Carlos III Health Institute (ISCIII), 28029 Madrid, Spain; 5 Department of Preventive Medicine and Public Health, Universidad de Zaragoza, 50009 Zaragoza, Spain

**Keywords:** acute myocardial infarction, care pathways, post-discharge follow-up, secondary prevention, gender differences, health services utilisation, real-world data

## Abstract

**Background/Objectives**: Secondary prevention after a first acute myocardial infarction (AMI) is crucial to reduce complications and improve long-term outcomes. Persistent gender inequalities in cardiovascular care suggest differences in post-AMI healthcare pathways between men and women. Understanding these trajectories could guide post-discharge clinical management, secondary prevention, and follow-up after acute myocardial infarction. This study aimed to describe healthcare pathways following a first AMI and explore gender inequalities within these trajectories. **Methods**: We conducted an observational, population-based study using real-world data (RWD) from the CARhES cohort. A total of 4298 individuals discharged alive after a first AMI between 2017 and 2022 were included. Healthcare trajectories during the 90 days following discharge were reconstructed across primary and specialised care, emergency services, and hospital admissions, and stratified by sex and the occurrence of clinical outcomes. **Results**: Post-AMI care pathways were highly heterogeneous. Although general practitioners often served as the first point of contact, most trajectories began in emergency departments. Patients with clinical outcomes showed higher healthcare utilisation. Women accessed primary care more frequently, whereas men showed greater use of specialised services and higher readmission rates, patterns that may reflect differences in follow-up strategies and clinical management during the early post-discharge period. Despite this variability, overall trajectories showed no significant sex-based differences. **Conclusions**: Post-AMI care pathways are complex and variable, with gender differences in patterns of service use. Women more often accessed scheduled care, while men relied more on emergency services. These findings highlight the need for gender-sensitive post-discharge follow-up to guide clinicians in secondary prevention strategies for AMI.

## 1. Introduction

Over the past several decades, cardiovascular disease (CVD) has been the leading cause of mortality on a global scale, with 20.5 million people dying each year from CVDs [1]. Within European countries, CVD accounts for 2.2 million deaths in females (47%) and 1.9 million deaths in males (39%) [2], with acute myocardial infarction (AMI) and strokes being the majority of CVD deaths [2,3]. In Spain, in 2023, 26.5% of deaths were due to diseases of the circulatory system. Ischaemic heart diseases were the most prevalent, constituting the primary cause of mortality among males (17,038 deaths) and the fourth leading cause among females (10,165 deaths) [4].

Healthcare following AMI, known as secondary prevention, plays a crucial role in reducing the recurrence of the event, decreasing morbidity and mortality, and enhancing cardiac function and quality of life [5,6,7]. Secondary prevention of AMI involves the initiation and adherence to cardioprotective medications, in conjunction with the effective management of cardiovascular risk factors (CVRFs) and the adoption of healthy lifestyle behaviours, as outlined in current European guidelines [6,8,9]. The early post-discharge period following an AMI represents a clinically vulnerable phase, during which follow-up planning, treatment optimisation, and secondary prevention strategies play a key role in patient management. Close follow-up, especially during the early post-discharge period and the first year following an AMI, is crucial to ensure appropriate use of healthcare resources, reinforce treatment adherence, and promote a collaborative patient–professional relationship, while also enabling clinical reassessment and adjustment of secondary prevention strategies that support recovery and long-term health maintenance [10].

Despite its importance, healthcare inequalities in the attention of CVD persist both between and within countries, with notable inequalities in CVD prevention and healthcare based on gender, ethnicity, or socioeconomic status, among others [1,11]. Recent studies have particularly drawn attention to gender inequalities in the healthcare and management of AMI [5,12,13,14]. According to the literature, women have lower access to cardiology visits than men [13,15] and less frequent monitoring and control of CVRFs [12,13,16,17]. While women engage more frequently with dietary and preventive measures, they are less likely to meet physical activity recommendations [13,18]. Furthermore, women are less frequently referred to cardiac rehabilitation after an AMI [5,13,19] and less often prescribed key guideline-recommended drugs for AMI [5,13,14,15,16,17].

These inequalities may persist during follow-up, influencing clinical monitoring, access to specialised care, and the intensity of secondary prevention strategies, and suggesting that post-AMI healthcare could differ by gender in ways that affect recovery and long-term outcomes [13,14]. We hypothesised that post-discharge healthcare pathways after a first AMI are heterogeneous and differ by gender, with potential implications for clinical follow-up and secondary prevention. To improve the quality and equity of post-AMI care, the objective of this study was to describe post-discharge healthcare pathways following a first episode of AMI and to explore gender-related differences with potential implications for clinical management and secondary prevention. Our findings highlight substantial variability in post-AMI care pathways and relevant gender differences in patterns of healthcare use during early follow-up.

## 2. Materials and Methods

### 2.1. Study Design and Population

This analytical study was designed to conduct a pathways analysis using observational, population-based data from the CARhES (CArdiovascular Risk Factors for hEalth Services research) cohort. CARhES is a population-based open and dynamic cohort established in the Spanish region of Aragon. It includes subjects aged 16 years and older who have been diagnosed with hypertension, diabetes mellitus, and/or dyslipidemia [20]. From a clinical perspective, this cohort allows the assessment of real-world post-AMI follow-up and healthcare trajectories across different levels of care. The study protocol was approved by the Clinical Research Ethics Committee of Aragon (CEICA PI21/148).

**Figure 1:**
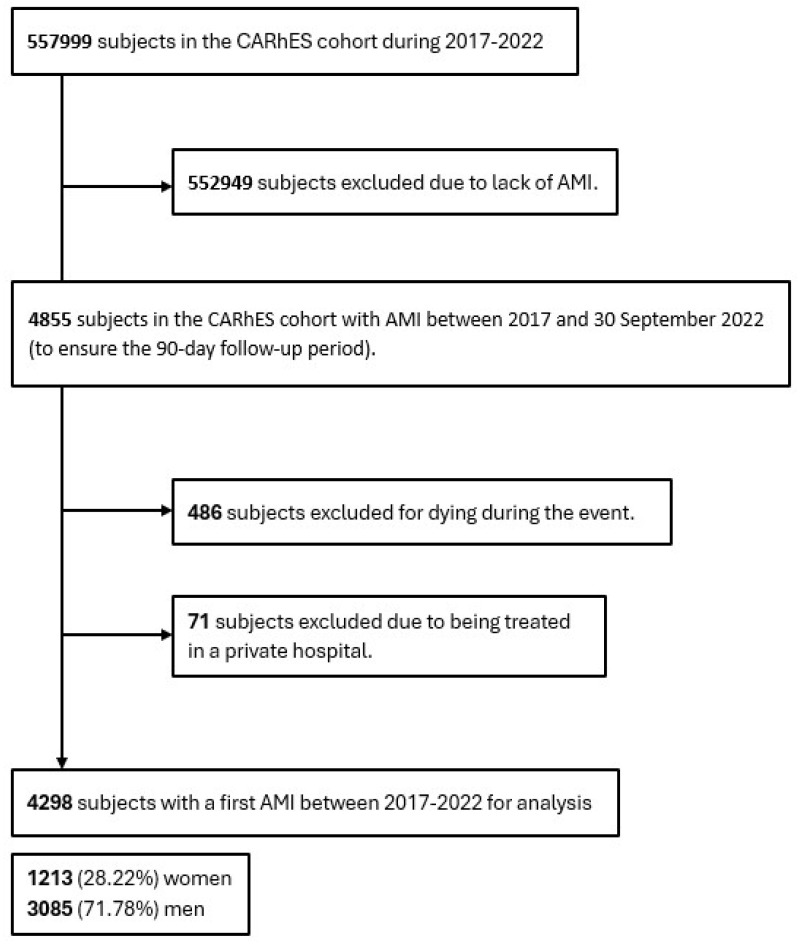
Study Flowchart: Overview of patient Inclusion and Exclusion process. AMI, acute myocardial infarction.

The study population were subjects experiencing a first AMI recorded in the CARhES cohort between 2017 and 30 September 2022, in order to ensure 90 days of follow-up. The presence of AMI was identified through the International Classification of Diseases (ICD-10) code I21. Subjects who had previously been diagnosed with an AMI at the onset of the cohort follow-up, or who died during the event, and those admitted and treated at private hospitals, were excluded from the study. Figure 1 presents a flowchart that illustrates the selection criteria of the study population.

### 2.2. Data Sources and Variables

The data used in this study were extracted from the CARhES cohort, which was constructed using real-world data (RWD) obtained from the electronic health records of the Aragon Health Service. This is a secondary analysis based on routinely collected healthcare data, capturing routine post-discharge clinical management after acute myocardial infarction across primary, emergency, and hospital care.

In this study, the variables were grouped into two main categories: general patient characteristics at the time of the AMI (including socio-demographic, anthropometric, and clinical variables), and pathway analysis variables, which included both healthcare services utilisation variables and health results for each patient.

### 2.3. General Patient Characteristics

At the time of the AMI, sociodemographic and anthropometric characteristics were collected: age, sex, nationality (categorised as native or immigrant), and place of residence (urban or rural, based on the basic healthcare area of residence of the patient). Data on the institutionalisation of the individual in a nursing home were also recorded. To determine socioeconomic status, income-related categories were employed, including pensioners with annual incomes below or above €18,000 (with or without access to free pharmacy benefits), unemployed individuals, employed individuals earning below or above €18,000 per year, and a residual category encompassing mutual insurance holders, those with special coverage, and individuals without insurance.

In the individuals where anthropometric measures were available, we included weight (kg) and height (cm), which were used to compute the body mass index (BMI), defined as weight in kilograms divided by height in metres squared. Classification of BMI was based on the World Health Organization (WHO) guidelines [21], with categories including underweight (BMI < 18.5 kg/m^2^), normal weight (18.5–24.9 kg/m^2^), overweight (25.0–29.9 kg/m^2^), and obese (BMI ≥ 30.0 kg/m^2^).

The existence of hypertension, diabetes mellitus, and dyslipidemia was systematically recorded.

Further clinical variables, such as comorbidities and morbidity burden, were obtained from the Morbidity-Adjusted Groups (GMA). GMA is a classification system that compiles diagnostic information from primary care, emergency departments, and hospital discharge records (Minimum Basic Data Set of Hospital Discharges) [22]. The system captures the major comorbid conditions for each individual, provides a numerical index of chronic disease burden, and includes indicators of clinical complexity.

### 2.4. Pathways Analysis Variables

In order to determine patients’ healthcare pathways during the follow-up, five different levels of care were considered: visits to a general practitioner (primary care), visits to a primary care nurse, specialist (restricted to cardiovascular-related specialties including cardiology, endocrinology and nutrition, cardiovascular surgery, nephrology, and ophthalmology), visits to the emergency department, and hospital admissions. Healthcare contacts were extracted from the day following the AMI, continuing for up to 90 days or until the patient exited the care pathway due to clinical outcomes (death or a new cardiovascular event). This 90 days follow-up period reflects the early post-discharge phase, which is critical for clinical reassessment and secondary prevention.

With regard to hospital admissions specifically, contacts were included only if they occurred after post-AMI hospital discharge. Repeated consecutive visits of the same type (e.g., multiple successive general practitioner visits) were consolidated into a single contact to avoid duplication in the trajectory diagrams. Furthermore, gender was considered a stratifying variable to enable disaggregated analysis of care pathways by gender. Three clinical outcomes, not mutually exclusive, were established during the 90-day follow-up: re-occurrence of AMI, death of the patient due to AMI, and death from any cause.

### 2.5. Statistical Analyses

Sociodemographic, baseline clinical and anthropometric characteristics of the study population were summarised using frequencies and percentages for categorical variables, and means with standard deviations (SD) for continuous variables. Gender comparisons were conducted using Pearson’s Chi-squared test for categorical variables and Student’s *t*-test for continuous variables. The threshold for statistical significance was set at *p* < 0.05.

Healthcare pathways were analysed using the bupaR package in R software (version 4.5.0), which transforms healthcare utilisation records into process-oriented trajectory diagrams. First of all, we explored which was the first healthcare contact of each subject after the AMI. Then, for each patient, we calculated his/her trace along the healthcare services within 90 days following an AMI. Each trace represents the ordered sequence of healthcare contacts that patients had, including any visits to the five levels of healthcare services considered. After that, we identified the most common pathways adopted by patients across the healthcare system during follow-up.

We obtained the diagrams of healthcare pathways. These diagrams consist of nodes and directed edges. Nodes represent individual healthcare activities (e.g., a visit to emergency care, hospitalisation, or outpatient services), and their size is proportional to the number of patients who have experienced that contact. The delineation of transitions between services is indicated by directional arrows, with the thickness of these arrows and the accompanying percentages reflecting the relative frequency of each transition. These diagrams offer a clear visual summary of the most frequent care sequences and the general structure of healthcare service use.

All these analyses were performed for the whole population of our study but also for men and women separately, in order to detect gender differences in the healthcare pathways of people after an AMI.

In addition, we selected those individuals of our population with an adverse clinical outcome (death or a new AMI) and analysed specifically their healthcare pathways, focusing on gender differences observed.

In order to examine whether the COVID-19 pandemic influenced post-AMI care patterns, a sensitivity analysis was conducted to compare the pre-COVID period (2017–2019) with the COVID period (2020–2022). Differences in the first point of healthcare contact and in the structure of the most frequent care pathways were assessed.

To further explore factors associated with healthcare utilisation after discharge, a multivariable logistic regression model was performed to analyse determinants of the type of first healthcare contact (scheduled visit versus emergency department). Independent variables included sex, age, socioeconomic status, residential area and comorbidities.

Finally, survival analyses were conducted to explore differences in adverse clinical outcomes during follow-up. Kaplan–Meier curves were used to compare event-free survival (death or recurrent AMI) between men and women, and a multivariable Cox proportional hazards model was fitted to identify factors associated with these outcomes. The outcome of interest was a composite of death or recurrent AMI during the 90-day follow-up period.

All statistical analyses were conducted using RStudio (version 2025.05.0 Build 496, Posit Software, Boston, MA, USA), R software (version 4.5.0, R Core Team 2022, R Foundation for Statistical Computing, Vienna, Austria) [23], and JAMOVI software (version 2.4, The Jamovi Project, Sydney, Australia) [Computer software], retrieved from https://www.jamovi.org [24].

No generative artificial intelligence tools were used in the design, analysis, or interpretation of the study.

## 3. Results

### 3.1. Study Population and Baseline Characteristics

In total, 4298 subjects who experienced a first AMI were included for analysis in our study. The study population comprised 1213 women (28.22%) and 3085 men (71.78%).

Sociodemographic and clinical characteristics of the study population are shown in Supplementary File Table S1. Women were significantly older than men at the time of AMI (76.46 vs. 68.20 years) and were more frequently institutionalised, and in lower socioeconomic groups. They also presented a greater burden of comorbidities and overall morbidity, particularly hypertension, depression, osteoporosis, and dementia (*p* < 0.001). In contrast, men showed higher rates of overweight and smoking (*p* < 0.001), although baseline characteristics differed significantly between sexes overall.

### 3.2. First Point of Contact in Post-Discharge Care

The patient’s first contact with the health care system after the AMI is shown in Table 1. A wide variability in the first point of access to the system can be observed. The most frequent initial contact was through emergency services (31.41%), followed by general practitioners (26.92%) and specialists (23.86%). Women were significantly more likely to first contact a specialist (25.99% vs. 23.03%) than men, but less likely to contact emergency services (28.63% vs. 32.50%). These results show multiple entry points into post-discharge care after AMI, with differences in initial access by gender.

Multivariable logistic regression identified four factors associated with the type of first healthcare contact after discharge (Table 2). Older age (OR 1.01; *p* = 0.004) and a diagnosis of depression (OR: 1.23; *p*: 0.030) increased the likelihood of a scheduled visit (general practitioner or specialist), while living in an urban area (OR 0.60; *p* < 0.001) and having prior ischemic heart disease (OR: 0.75; *p*: 0.012) were associated with greater use of the emergency department as the first point of contact.

Sex was not independently associated with the type of first healthcare contact after adjustment for the remaining variables. However, exploratory analyses stratified by sex suggested that the factors associated with healthcare utilisation may differ slightly between men and women. In particular, comorbidities appeared to influence the probability of scheduled versus emergency contact among men, whereas no clear associations were observed among women. These results are presented in the Supplementary File (Table S2).

### 3.3. Post-Discharge Care Pathways During the First 90 Days

**Figure 2:**
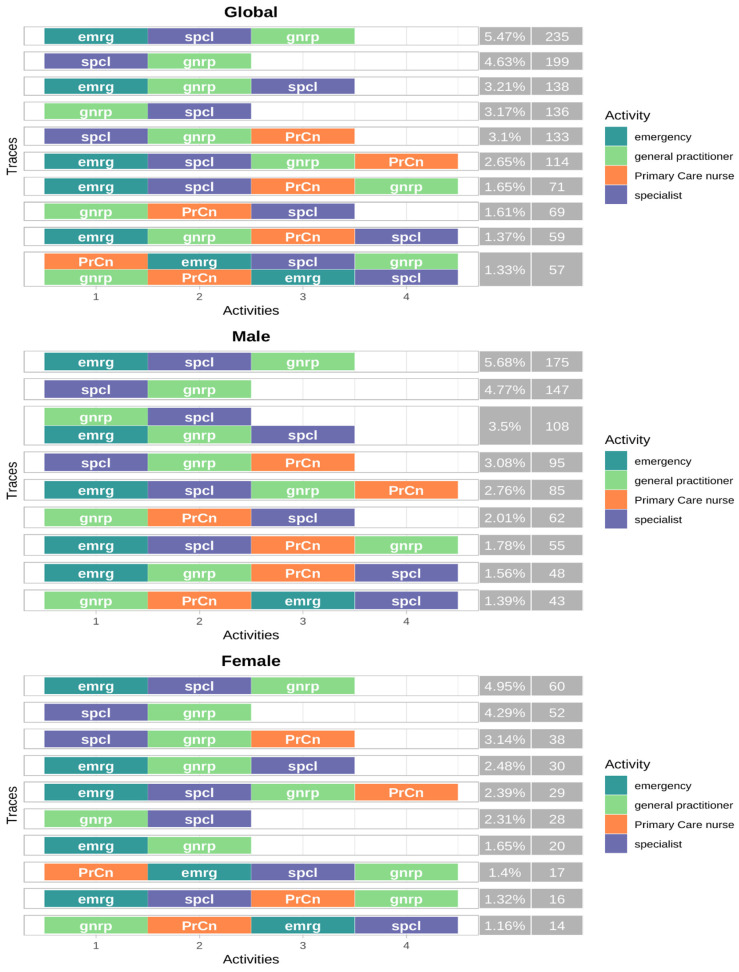
Post-discharge care pathways during the first 90 days after acute myocardial infarction, overall and stratified by gender. The figure shows the ten most frequent post-discharge care pathways observed in the overall study population and separately for women and men. Pathways represent ordered sequences of healthcare contacts during the 90-day follow-up period.

Analysis of health care pathways following AMI (Figure 2) revealed substantial heterogeneity across the whole population. Emergency services were the predominant first contact, featuring in five of the ten most common sequences, while primary care initiated only three. The most frequent pathway combined emergency care, specialist consultation, and GP visit (5.47%), followed by specialist then GP contact (4.63%). Notably, primary care nursing was absent from all four top trajectories throughout the 90-day follow-up.

A large number of distinct care sequences were observed during the 90-day follow-up period.

Differential analysis by gender, showed that the two most frequent pathways were common in both groups, indicating the presence of shared patterns of care. For both men and women, the most frequent pathway started with a visit to an emergency department, followed by a specialist and a general practitioner. Nonetheless, this pathway was slightly less frequent among women (5.68% vs. 4.95%). Furthermore, five trajectories among the top ten initiated through emergency services, and two with specialists. Notably, men used primary care nursing services slightly more often than women across their top ten pathways (six pathways vs. five in women). However, only women initiated contact through a primary care nurse, highlighting a potentially distinct first contact with the healthcare system among female patients.

Overall, the distribution of the most frequent pathways showed similarities between genders, with some differences in the use of specific services.

To facilitate interpretation, the following figure summarises the most frequent healthcare pathways observed during the 90-day follow-up period, overall and by gender (Figure 2).

To determine whether the pandemic influenced patterns of healthcare access, a sensitivity analysis was conducted, comparing the pre-COVID period (2017–2019) and the COVID period (2020–2022). Similar patterns in the first point of contact were observed. Emergency departments remained the most frequent first access (31.86% vs. 29.67%), followed by general practitioners and specialists (Supplementary File Table S3). The most frequent care trajectories also remained stable. Nine of the top ten pathways were observed both in the pre- and post-COVID-19 period, suggesting that the pandemic did not substantially modify the patterns of post-AMI healthcare utilisation (Supplementary File Figure S1). A total of 345 distinct care pathways were needed to cover 80% of the population, reflecting considerable complexity in post-AMI healthcare navigation, with similar levels observed in both men (288 unique pathways) and women (292).

**Figure 3:**
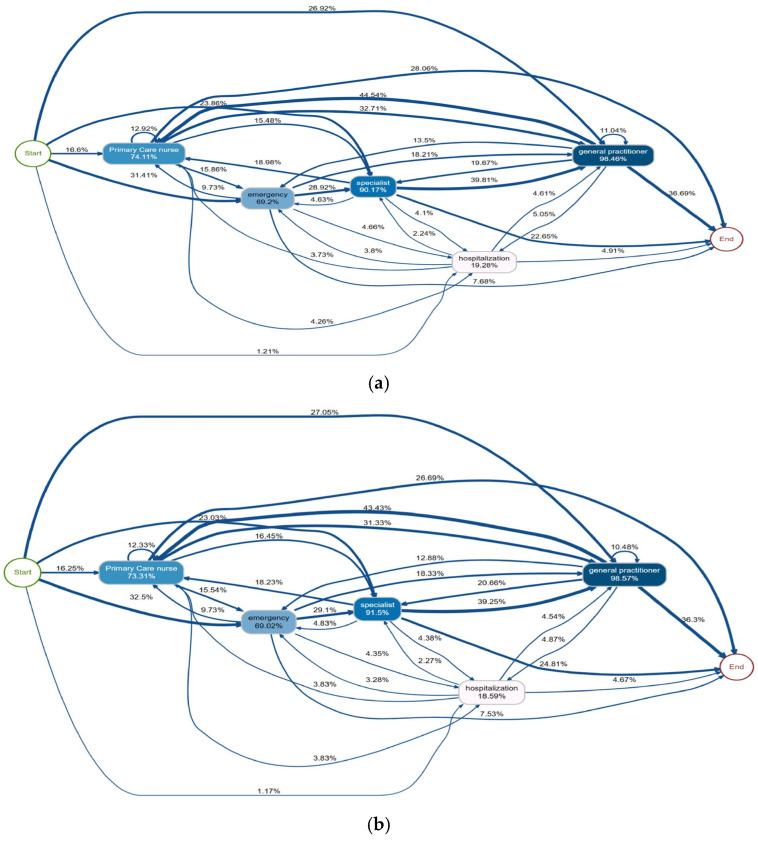

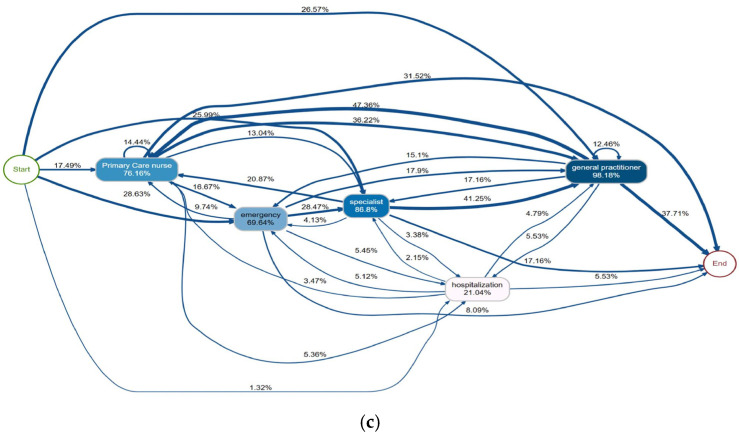
Post-discharge care pathway process maps during the first 90 days after acute myocardial infarction. (**a**) Overall population; (**b**) Men; (**c**) Women. Node size is proportional to the number of patients with at least one contact at each level of care, and arrow thickness reflects the frequency of transitions between services during follow-up.

In the process map representing the complete care pathways (Figure 3), the initial point, designated ‘Start’, corresponds to the patient’s first point of contact with the healthcare system. The arrow connecting “Start” to a specific node represents the percentage of patients whose initial visit was to that service, as reported in Table 2. Each node indicates the proportion of patients that had, at least, one visit to the corresponding service. The most commonly visited service was the general practitioner (98.46%), followed by specialist consultations (90.17%) and primary care nurse (74.11%). The arrows in the diagram represent the directional transitions between services, illustrating the sequential order in which patients navigated through the healthcare system. Common sequential transitions included specialist attendance after emergency care (28.92%), general practitioner after specialist (39.81%), and primary care nursing as a subsequent step (44.54%).The final node, designated ‘End’, denotes the patient’s exit from the care pathway, which corresponds to death, the occurrence of a new cardiovascular event or the completion of the 90-day follow-up period. Exit from the pathway most commonly followed a general practitioner visit (36.69%) or a nursing consultation (26.92%).Minor gender differences were observed: women accessed primary care nursing more frequently (76.16% vs. 73.31%), while men had higher specialist utilisation (91.50% vs. 86.80%). Overall, pathway diversity appeared largely independent of gender.

### 3.4. Post-Discharge Care Pathways Among Patients Experiencing Clinical Outcomes

During the 90-day follow-up, 352 patients (8.19%) experienced at least one clinical outcome: 223 men (7.23%) and 129 women (10.63%). Sociodemographic and clinical characteristics of these patients are presented in Supplementary File Table S4. The incidence of clinical outcomes stratified by gender is available in Supplementary File Table S5.

A multivariable Cox proportional hazards regression model was fitted to identify independent predictors of death or recurrent AMI (Table 3). After adjustment for the remaining variables, sex was not independently associated with the risk of adverse outcomes (HR 1.00; 95% CI 0.78–1.28). Increasing age (HR 1.04; 95% CI 1.03–1.05), diabetes mellitus (HR 1.34; 95% CI 1.08–1.67), chronic kidney disease (HR 1.36; 95% CI 1.07–1.72), and dementia (HR 1.49; 95% CI 1.00–2.20) were associated with a higher risk of adverse outcomes.

Kaplan–Meier survival curves showed a lower probability of event-free survival (death or recurrent AMI) among women compared with men during follow-up (log-rank *p* = 0.00036) (Supplementary File Figure S2).

**Figure 4:**
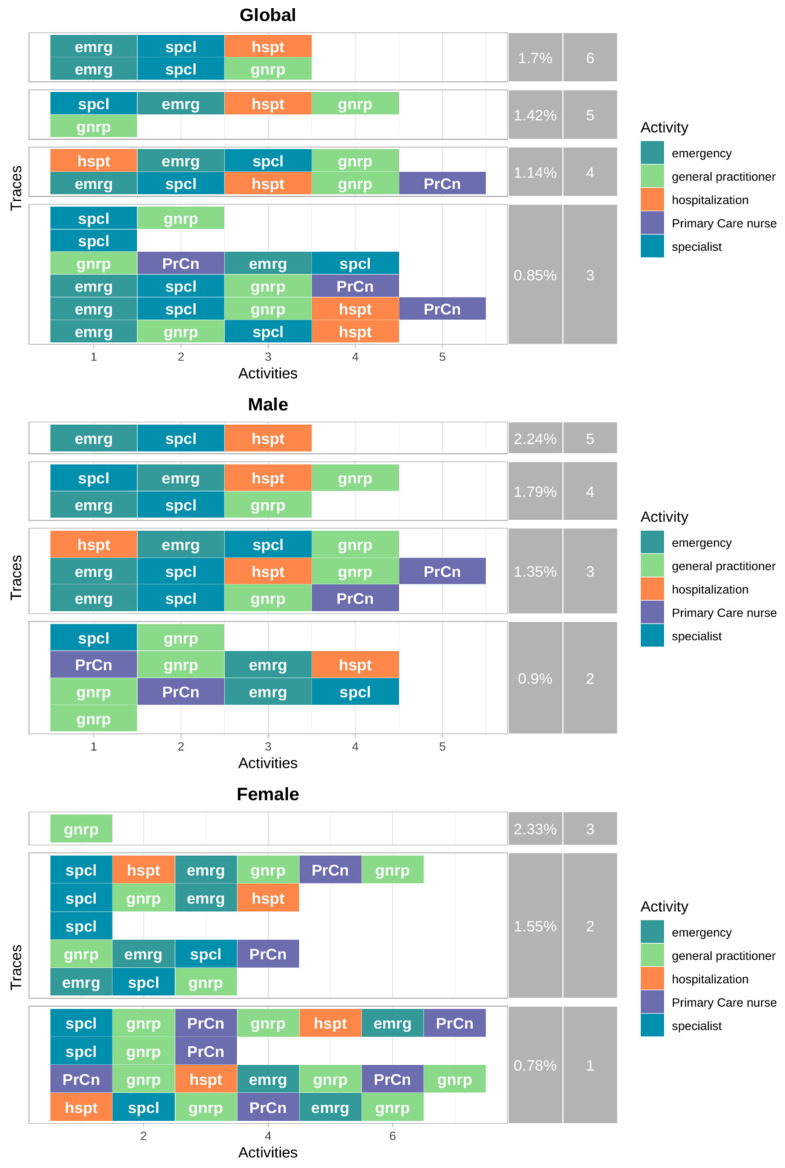
Post-discharge care pathways among patients experiencing clinical outcomes within 90 days after acute myocardial infarction, stratified by gender. The figure displays the ten most frequent care pathways observed during follow-up among patients who experienced recurrent acute myocardial infarction or death. Pathways are shown separately for the overall population, men, and women.

Average follow-up time and the distribution of initial healthcare contacts among patients with clinical outcomes are presented in the Supplementary File (Tables S6 and S7). Follow-up duration was similar between men and women, with no statistically significant differences. General practitioners were the most frequent initial point of contact, followed by specialists and emergency services, with some gender differences observed in the pattern of access. The analysis of the most frequent healthcare pathways for patients who had clinical outcomes (Figure 4) revealed significant variability, with only 42 subjects within the 10 most frequent pathways. Five of the top ten pathways initiated through emergency services, confirming their role as the predominant point of first contact, while only two started in primary care or specialist settings. The most frequent trajectories involved emergency care followed by specialist consultation and hospitalisation or primary care (1.7% each). Gender differences were observed, with men more frequently initiating care through emergency services (four vs. one pathways), whereas women more often started through specialists (five vs. two) and showed slightly higher use of primary care nursing services.

Process maps representing the complete post-discharge care pathways among patients experiencing clinical outcomes are shown in Supplementary File Figure S3. General practitioners (94.89%), emergency services (84.84%), and specialist consultations (82.39%) were the most frequently visited healthcare levels, with recurrent transitions between primary care and nursing services. Overall patterns were similar between men and women, although some differences in service utilisation were observed.

## 4. Discussion

This study reveals complex and heterogeneous healthcare utilisation after a first AMI, with several gender-based differences. Women and men have different sociodemographic and clinical profiles after a first AMI. Their first contact with the healthcare system differs, as do their subsequent care pathways and trajectories. These findings are consistent with our working hypothesis that post-discharge care after AMI is highly variable and may differ by gender, with potential implications for clinical follow-up and secondary prevention strategies.

Women in our population were significantly older, more frequently institutionalised, and disproportionately represented in lower income groups. On a clinical level, women also exhibit higher levels of comorbidity and morbidity burden. These characteristics are clinically relevant, as age, multimorbidity, and socioeconomic vulnerability are well-established key determinants of follow-up needs, treatment adherence, prognosis after AMI, and patterns of healthcare utilisation, which may contribute to inequalities in access to and continuity of care following AMI [25,26,27,28,29]. Age-related barriers, such as underrecognition of symptoms, diagnostic biases, or lack of prioritisation of older adults in clinical decision making, have been associated with poorer access to timely and appropriate care, particularly among women [29,30]. In addition, individuals with lower incomes or residing in areas with lower socioeconomic status are less likely to have access to specialised healthcare services or adequate health follow-up [28,31,32]. Furthermore, patients with higher morbidity have more challenging care trajectories and less follow-up in specialised settings, despite their greater clinical complexity and health needs [27,30].

Regarding the initial point of contact with the health system, in line with existing evidence [33,34], our study found that emergency departments and general practitioners were the most common initial points of contact within the health system. Several qualitative studies have explored the reasons behind patients’ choice of frontline services. In the case of emergency department visits, patients often seek care due to the sudden onset or worsening of symptoms, the perception that they need more immediate care, or because they cannot access primary care appointments in a timely manner [35,36,37]. Additionally, Wang et al. identified factors associated with repeated emergency department use after hospital discharge, including functional limitations (e.g., difficulty walking), polypharmacy, unmet care needs, and insufficient or unclear discharge planning information, which disproportionately affect older adults and highlight the clinical importance of effective discharge planning and early follow-up to reduce unplanned emergency care after AMI [38].

Conversely, patients with a well-established relationship with their general practitioner, characterised by continuity of care and trust in the physician’s ability to manage their condition, are more likely to initiate follow-up through primary care services [35,36,37]. Primary care–led follow-up may represent a more structured and coordinated approach to secondary prevention, as it often marks the initiation of systematic risk factor control, medication optimisation, and lifestyle modifications counselling [10,39].

In addition, the multivariable analysis conducted in this study suggests that the type of first healthcare contact after discharge is influenced by clinical and contextual factors such as age, residential area and certain comorbidities. Sex was not independently associated with the probability of initiating care through scheduled visits or emergency departments after adjustment for these variables. The relatively limited discriminative capacity of the model also suggests that additional organisational or contextual factors not captured in the available data may contribute to patients’ healthcare-seeking behaviour [35,36,37].

When stratified, gender disparities were observed: men were more likely to initiate care in the emergency department, while women did so in the specialist. This finding is of particular interest, given that women in the cohort exhibited higher rates of reinfarction (7.01% vs. 5.12%) and mortality (4.78% vs. 2.85%) during follow-up. However, the multivariable survival analysis conducted in this study suggests that these differences cannot be explained by sex alone. After adjustment for clinical and sociodemographic characteristics, sex was not independently associated with the risk of death or recurrent AMI within 90 days after discharge, whereas age and several comorbidities showed stronger associations with adverse outcomes. The commonly held assumption that men visit emergency services more frequently due to a higher risk of reinfarction or greater complications does not hold in this context. These patterns suggest that emergency department use may not accurately reflect underlying clinical risk. Instead, behavioural and clinical factors may underlie this observation. Men with typical cardiac symptoms may have a greater awareness of cardiovascular risk and be more likely to perceive their condition as urgent [40,41]. Women, conversely, often report atypical symptoms and minimise them, which can result in delayed recognition, underestimation of risk, and failure to prioritise their care. Consequently, women may choose to delay seeking medical attention until their scheduled visit and consult primary care rather than seeking emergency care [41,42,43].

Our study revealed a high degree of heterogeneity in care trajectories after a first AMI, with more than 340 different pathways identified. This variability is remarkable given the existence of structured clinical protocols and guideline-based recommendations for post-AMI care and highlights the absence of a dominant or standardised follow-up pathway in routine clinical practice.

The pathway diagrams provide a visual summary of the most frequent healthcare trajectories, although they necessarily simplify the complexity of individual patient journeys. The variability observed likely reflects both patient characteristics and organisational factors within the healthcare system.

Despite the presence of similarities in the initial contacts, directions and endpoints, no single pathway dominated the healthcare system. The observed heterogeneity does not appear to be influenced by gender or clinical necessity, but rather by patient-initiated demand, particularly in services with less restrictive access, such as general medicine, emergency departments, and nursing care [35,38,39]. Other factors such as waiting lists or accessibility of some medical services may also play a part [28,32,44], including contextual circumstances such as the COVID-19 pandemic, although no substantial differences were observed in our cohort.

Factors such as the perceived severity of symptoms, trust in healthcare providers, previous experiences or contextual elements (such as place of residence or availability of services) could have also influenced care-seeking behaviour [32,34,35]. Although some trajectories were common across both genders, notable disparities emerged: men were more likely to follow trajectories with emergency services or specialty care, whereas women attended more frequently nursing services. These patterns may reflect gender differences in health-seeking behaviour and perceptions of healthcare roles, influenced by social norms, symptom appraisal, and prior experiences with the healthcare system. Men tend to seek more direct or urgent care and are less likely to engage with primary care, partly due to male behaviours such as stoicism and self-reliance, as well as structural barriers like limited clinic hours or long waiting times. In contrast, women tend to prioritise continuity and relational care [10,44].

Patients with clinical outcomes had a higher utilisation of healthcare services during the follow-up period. In particular, they showed higher rates of reconsultation, especially among female patients, and an increased number of hospitalisations compared to their subjects without adverse clinical outcomes (79.55% vs. 19.28%). While the most frequent initial point of contact were general practitioners, the majority of care trajectories started in emergency departments. As observed among the general population, there was substantial variability in care pathways, but no major structural differences appeared in pathway maps or transition patterns.

Gender-based disparities were observed, with women showing a tendency to have more frequent primary care consultations, while men presented higher rates of hospital readmissions and rehospitalisations. One plausible explanation for this discrepancy is the higher short-term mortality observed among women, which may reduce the likelihood of subsequent hospitalisation if complications are fatal. As previous studies have shown, being female is an independent predictor of cardiovascular mortality [45], and women also have lower rates of hospital admission and rely more on primary care services [6]. Furthermore, a gender bias in the diagnosis and treatment of reinfarction cannot be ruled out. Evidence suggests that women are less likely to be referred for coronary angiography or admission to intensive care units, and they often receive percutaneous coronary intervention less frequently than men [46,47,48]. This may lead to under-treatment and under-hospitalisation. Additionally, women tend to rely more on primary care [13,26], which may reflect atypical symptom presentation and structural barriers to specialist care.

Finally, it is important to highlight that the average duration of follow-up was similar across all patients in the cohort, including those who experienced clinical outcomes. Consequently, the observed differences in care pathways are not related with the time of follow-up, and could be attributable to individual-level factors such as age, gender, multimorbidity, socioeconomic status, educational attainment, and health beliefs and structural factors, such as healthcare organisation and waiting lists. All these factors influence care-seeking behaviours and access to healthcare services [27,28,30,32]. Future research could benefit from incorporating qualitative methodologies, among others, to better understand the determinants of pathway diversity.

### Strengths and Limitations

A key strength of this study is the use of the CARhES cohort, a large, population-based dataset obtained from routinely collected RWD within the Aragon Health Service. The high-quality electronic health records used in this study provide a representative regional sample, thereby enhancing both the internal validity and generalisability of the findings. The substantial sample size and the incorporation of data from multiple care settings—primary care, specialist consultations, emergency services and hospitalisations—facilitate a comprehensive and detailed evaluation of the healthcare response in a post-discharge period. Another strength is the application of pathway and trajectories analysis, which provides a clinically meaningful framework to visualise and understand patient trajectories across levels of care, offering both objective and visual insights into patients’ post-AMI journeys across different levels of the healthcare system. This method captures the complexity and variability of healthcare use, helping to identify the most and least utilised services. These findings are useful to design clinical pathways with the objective of promoting more coordinated and efficient post-AMI care. Furthermore, the gender-stratified analysis facilitates the identification of inequalities in care access and utilisation, thereby providing valuable evidence to enable more equitable and gender-sensitive healthcare planning.

However, some limitations should be acknowledged. As with all studies based on routinely collected clinical data, there is a potential for diagnostic misclassification or underdiagnosis, particularly among women, whose symptoms may be less easily recognised in clinical settings. Furthermore, in order to facilitate the analysis of pathways, consecutive visits within the same level of care were consolidated into a single contact. This approach facilitates the identification of trajectories but limits the assessment of visit frequency and the identification of high users or “super-utilisers” of healthcare services. However, this approach was appropriate given the study’s objective of describing care pathways’ structure rather than intensity.

The study also lacked information on the specific reason for each healthcare visit, whether to primary care or the emergency department. While the proximity of these contacts to the AMI event suggests a likely relationship, this cannot be confirmed with certainty. In addition, information on participation in cardiac rehabilitation programmes was not available in the electronic health records used for this study. As cardiac rehabilitation is a key component of secondary prevention after AMI, the absence of these data may limit the interpretation of post-discharge healthcare utilisation patterns. Finally, care pathways were not associated with health outcomes due to the high heterogeneity of trajectories and the relatively low number of adverse clinical outcomes. Therefore, the effectiveness of care patterns could not be evaluated. Exploration of this aspect in future studies with larger populations, a higher number of clinical outcomes, or longer follow-up periods would be of high interest.

## 5. Conclusions

This study illustrates the significant heterogeneity and complexity of the diverse post-discharge care pathways followed by post-AMI patients. Women with AMI in our population were found to be older, to have a lower socioeconomic status and a greater number of comorbidities.

Women accessed health services mainly through scheduled visits (specialists or primary care), whereas men primarily used the emergency services. Patients who suffered adverse clinical outcomes had a greater utilisation of health services, including higher rates of hospital readmission and reconsultation, particularly among women. Gender differences in patterns of care were also observed, with greater use of specialised services among men and greater reliance on primary care among women. These patterns may be indicative of gender-based disparities in disease progression and clinical management and follow-up, as well as other personal and structural factors playing a key role in the care attention received. Such factors may include traditional gender roles influencing health-seeking behaviour, differences in symptom perception and reporting, the severity and presentation of clinical symptoms, the nature of the doctor–patient relationship in primary care, and levels of trust in healthcare providers, among others. Structural determinants such as place of residence (e.g., rural versus urban settings), socioeconomic status, and differential access to specialised services may also contribute to these trajectories.

Overall, these findings highlight the absence of structured and systematic follow-up care after AMI, with care trajectories driven more by acute episodes than by routine post-discharge management, despite the existence of clinical guidelines. It is therefore necessary to design structured and systematic gender-sensitive follow-up strategies after AMI to reduce care variability and improve continuity of care, especially in primary care. Strengthening referral systems and personalising follow-up strategies for complex patients, irrespective of gender, is essential. Standardising care trajectories, along with exploring patient experience and behaviour, could help identify modifiable barriers, while integrated care teams and more flexible hours in healthcare attention may also reduce inequalities and optimise secondary prevention.

## Figures and Tables

**Table 1:** First point of contact in post-discharge care after AMI, overall and stratified by gender.

N, %	Overall		Women		Men		*p* Values
General practitioner	1156	26.92	322	26.57	834	27.05	0.7476
Primary Care nurse	713	16.60	212	17.49	501	16.25	0.3251
Specialist	1025	23.86	315	25.99	710	23.03	**0.0405**
Emergency	1349	31.41	347	28.63	1002	32.50	**0.01391**
Hospitalisation	52	1.21	16	1.32	36	1.17	0.681

AMI: acute myocardial infarction. N: number %: percentage. *p*: statistical significance *p* < 0.05. Pearson’s Chi-squared test. Bold values indicate statistical significance (*p* < 0.05).

**Table 2:** Multivariable logistic regression analysis of factors associated with the type of first contact after AMI (scheduled visit versus emergency department).

Characteristic	OR	95% CI	*p*-Value
**Gender**			
Women (Ref. Men)	1.09	0.93, 1.28	0.3
**Age**	1.01	1.00, 1.01	**0.004**
**Socioeconomic status**			
≥18,000 € per year (Ref. < 18,000 € per year)	0.90	0.78, 1.04	0.14
Residential area			
Urban (Ref. Rural)	0.60	0.52, 0.70	**<0.001**
**Comorbidities**			
Hypertension	1.01	0.87, 1.18	>0.9
Dyslipemia	1.15	0.72, 1.79	0.6
Diabetes Mellitus	0.95	0.84, 1.09	0.5
Overweight	0.89	0.74, 1.07	0.2
Ischemic heart disease	0.75	0.60, 0.94	**0.012**
Chronic Obstructive Pulmonary Disease	0.93	0.75, 1.16	0.5
Depression	1.23	1.02, 1.49	**0.030**
Chronic Kidney Disease	0.89	0.76, 1.05	0.2
Cirrhosis	0.88	0.63, 1.25	0.5
Dementia	0.99	0.69, 1.46	>0.9

Ref = reference category, OR = Odds Ratio, CI = Confidence Interval, *p*: statistical significance *p* < 0.05. Bold values indicate statistical significance (*p* < 0.05).

**Table 3:** Multivariable Cox proportional hazards model for predictors of death or recurrent acute myocardial infarction within 90 days after discharge.

Risk Factor	HR	95% CI	*p*-Value
**First Visit**			
Scheduled visit (Ref. Emergency)	1.37	1.06, 1.77	**0.014**
**Gender**			
Women (Ref. Men)	1.00	0.78, 1.28	>0.9
**Age**	1.04	1.03, 1.05	**<0.001**
**Socioeconomic status**			
≥18,000 € per year (Ref. < 18,000 € per year)	0.91	0.70, 1.17	0.5
Residential area			
Urban (Ref. Rural)	1.07	0.85, 1.36	0.6
**Comorbidities**			
Hypertension	0.85	0.66, 1.10	0.2
Dyslipemia	0.54	0.36, 0.81	**0.003**
Diabetes Mellitus	1.34	1.08, 1.67	**0.008**
Overweight	1.06	0.78, 1.43	0.7
Ischemic heart disease	0.31	0.25, 0.40	**<0.001**
COPD	1.14	0.83, 1.57	0.4
Depression	0.91	0.68, 1.22	0.5
Chronic Kidney Disease	1.36	1.07, 1.72	**0.010**
Cirrhosis	0.72	0.36, 1.47	0.4
Dementia	1.49	1.00, 2.20	**0.047**

HR: Hazard Ratio; CI: Confidence Interval; *p*: statistical significance *p* < 0.05; Ref.: reference category; COPD: Chronic Obstructive Pulmonary Disease. Bold values indicate statistical significance (*p* < 0.05).

## Data Availability

The data supporting the findings of this study are derived from the CARhES cohort and are not publicly available due to legal and ethical restrictions related to sensitive health information and data protection regulations. However, the research team is open to scientific collaborations aligned with the main objectives of the cohort. Researchers interested in conducting data analyses, developing new methodological approaches, or performing cross-national comparisons may contact the Principal Investigators of the cohort: Isabel Aguilar-Palacio (iaguilar@unizar.es) and Sara Malo (smalo@unizar.es).

## Supplementary Materials

The following supporting information can be downloaded at: https://www.mdpi.com/article/10.3390/jcm15072592/s1, Table S1. Baseline sociodemographic and clinical characteristics of patients with a first AMI, overall and stratified by gender. Table S2. Multivariable logistic regression analysis of factors associated with the type of first contact after AMI (scheduled visit versus emergency department), stratified by sex. Table S3: First point of contact in post-discharge care after AMI before and during the COVID-19 pandemic. Table S4. Baseline sociodemographic and clinical characteristics of patients experiencing clinical outcomes after acute myocardial infarction, overall and stratified by gender. Table S5: Incidence of clinical outcomes within 90 days after acute myocardial infarction, stratified by gender. Table S6: Average follow-up time (in days) after acute myocardial infarction, overall and stratified by gender. Table S7: First point of contact in post-discharge care among patients experiencing clinical outcomes after acute myocardial infarction, stratified by gender. Figure S1: Comparison of the most frequent post-AMI healthcare pathways in the pre-COVID (2017–2019) and COVID (2020–2022) periods. Figure S2: Kaplan–Meier curves for event-free survival (death or recurrent acute myocardial infarction) within 90 days after discharge, stratified by sex. Figure S3: Post-discharge care pathway process maps among patients experiencing clinical outcomes within 90 days after acute myocardial infarction. (a) Overall population; (b) Men; (c) Women.

## Abbreviations

The following abbreviations are used in this manuscript:

AMI	Acute Myocardial Infarction
BMI	Body Mass Index
CARhES	CArdiovascular Risk factors for hEalth Services research (cohort)
CEICA	Comité Ético de Investigación Clínica de Aragón
CVD	Cardiovascular Disease
CVRF	Cardiovascular Risk Factors
GMA	Grupos de Morbilidad Ajustados (Morbidity-Adjusted Groups)
ICD-10	International Classification of Diseases, 10th revision
N	Number
*p*	Statistical significance (*p* < 0.05)
RWD	Real-World Data
SD	Standard Deviation
WHO	World Health Organization
%:	Percentage
