# Supplementary material for: Care Pathways After Acute Myocardial Infarction: A Gender-Based Perspective
Source: J Clin Med. 2026 Mar 28;15(7):2592. doi: 10.3390/jcm15072592 (PMC13073914; doi:10.3390/jcm15072592)

Figure S1. Comparison of the most frequent post-AMI healthcare pathways in the pre-COVID (2017–2019) and COVID (2020–2022) periods.

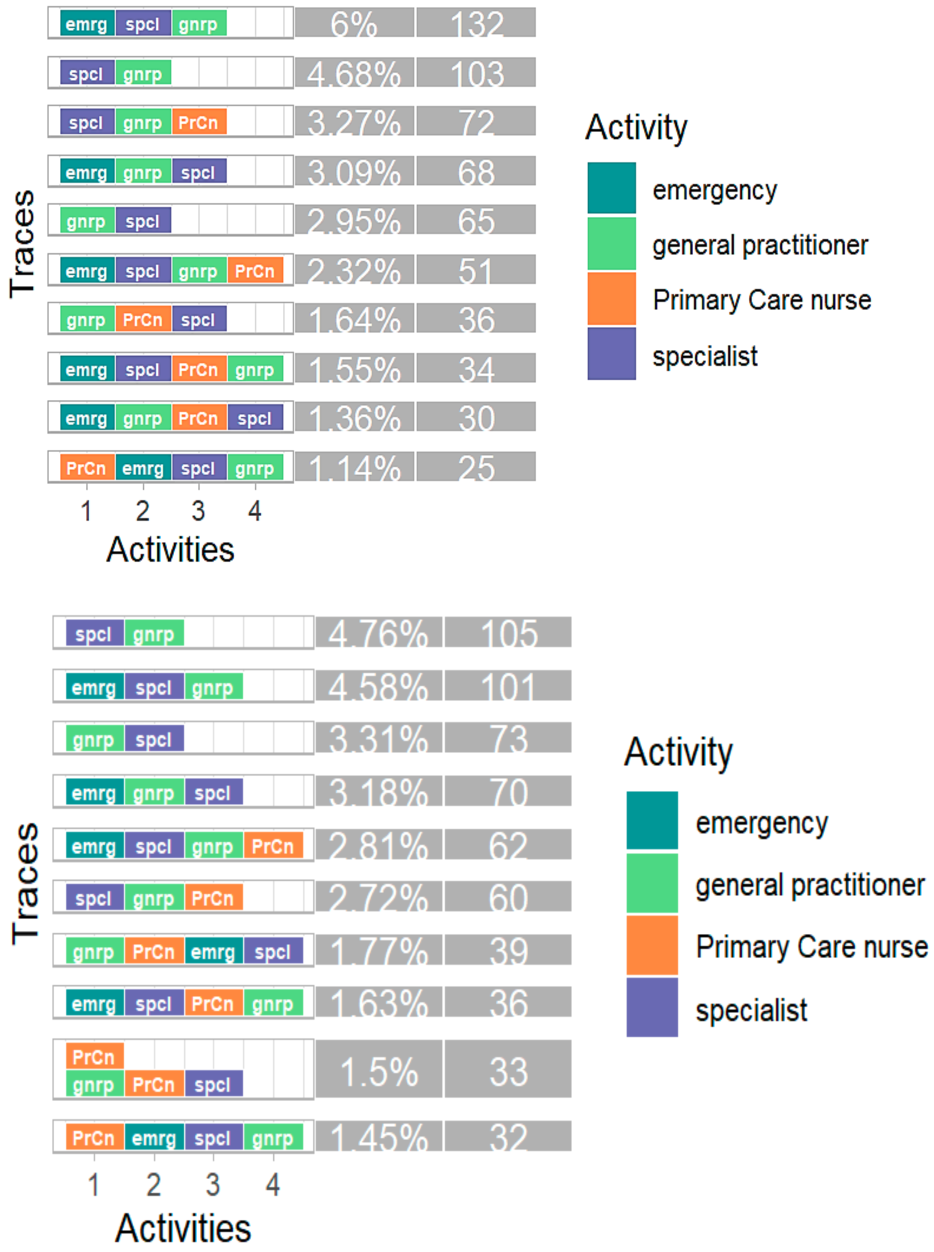

Supplement: Supplementary file 1 [file jcm-15-02592-s001.zip › Figure S1.pdf]
