# Supplementary material for: Care Pathways After Acute Myocardial Infarction: A Gender-Based Perspective
Source: J Clin Med. 2026 Mar 28;15(7):2592. doi: 10.3390/jcm15072592 (PMC13073914; doi:10.3390/jcm15072592)

Figure S2. Kaplan–Meier curves for event-free survival (death or recurrent acute myocardial infarction) within 90 days after discharge, stratified by sex.

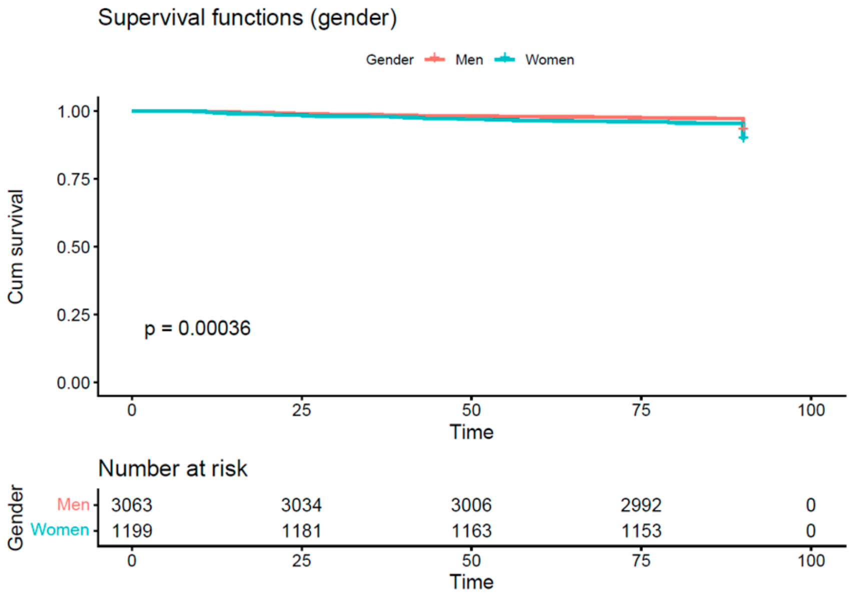

Supplement: Supplementary file 1 [file jcm-15-02592-s001.zip › Figure S2.pdf]
