# Supplementary material for: Care Pathways After Acute Myocardial Infarction: A Gender-Based Perspective
Source: J Clin Med. 2026 Mar 28;15(7):2592. doi: 10.3390/jcm15072592 (PMC13073914; doi:10.3390/jcm15072592)
Supplement: Supplementary file 1 [file jcm-15-02592-s001.zip › Figure S3..pdf]

Figure S3. **Post-discharge care pathway process maps among patients experiencing clinical outcomes within 90 days after acute myocardial infarction.**
(a) Overall population; (b) Men; (c) Women.
Node size is proportional to the number of patients with at least one contact at each level of care, and arrow thickness reflects the frequency of transitions between services during follow-up.

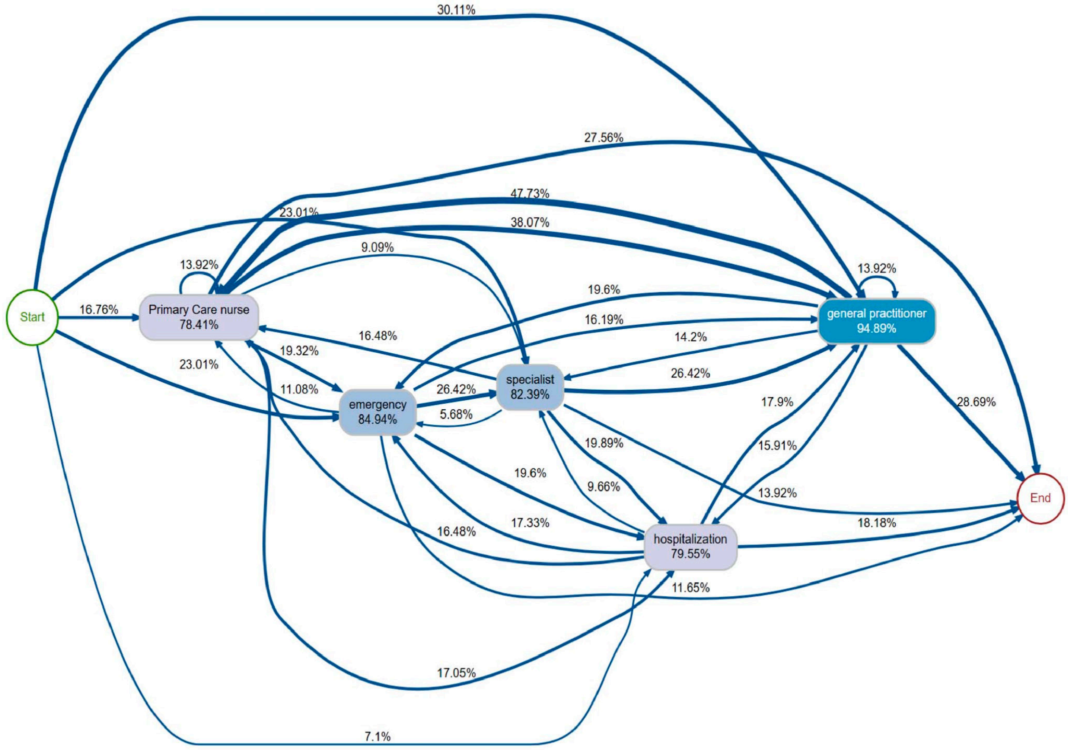

(a)

(b)

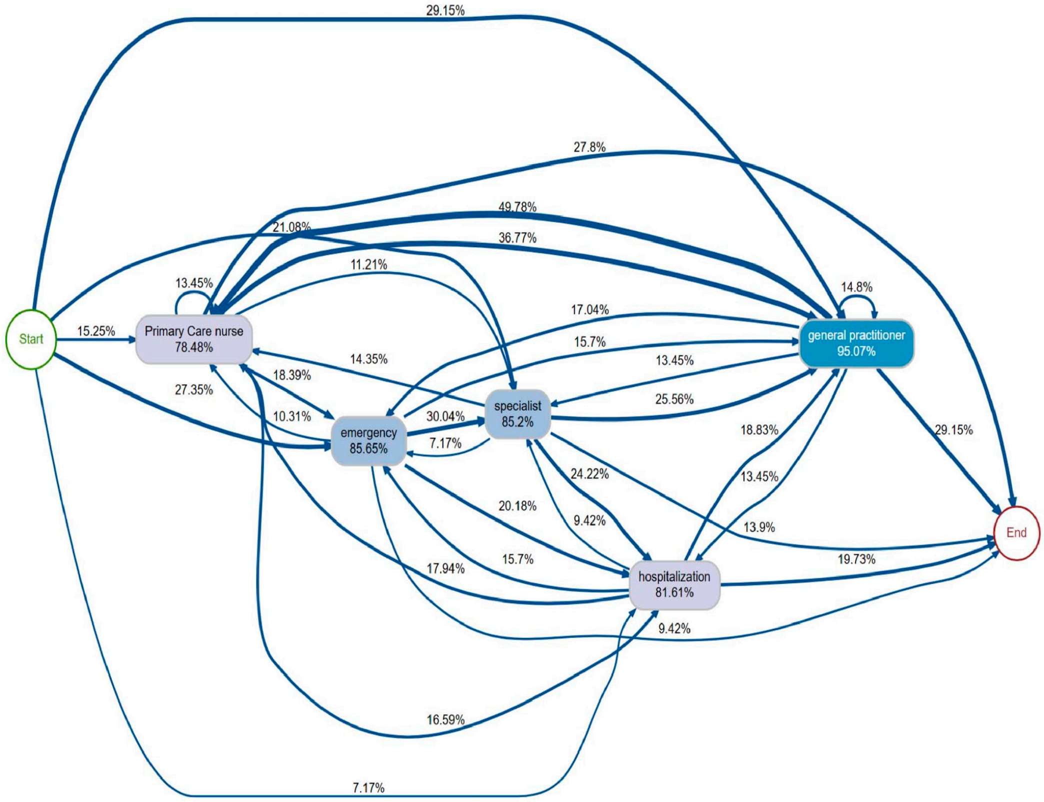
29.15%
27.8%
49.78%
21.08%
36.77%
11.21%
13.45%
14.8%
Start
15.25%
Primary Care nurse
78.48%
17.04%
15.7%
general practitioner
95.07%
14.35%
13.45%
18.39%
27.35%
specialist
85.2%
25.56%
10.31%
30.04%
emergency
85.65%
7.17%
29.15%
18.83%
24.22%
13.45%
20.18%
9.42%
13.9%
End
17.94%
15.7%
19.73%
hospitalization
81.61%
9.42%
16.59%
7.17%

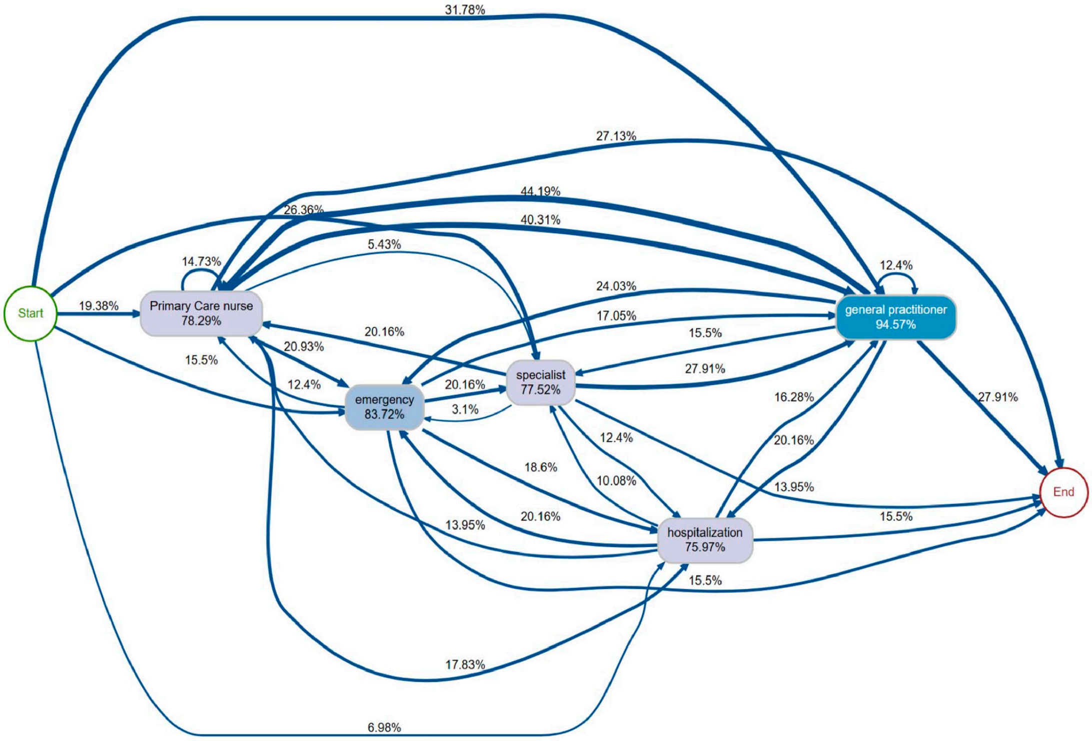
31.78%
27.13%
44.19%
26.36%
40.31%
5.43%
14.73%
12.4%
24.03%
Start
19.38%
Primary Care nurse
78.29%
general practitioner
94.57%
17.05%
20.16%
15.5%
15.5%
20.93%
27.91%
12.4%
20.16%
specialist
77.52%
emergency
83.72%
3.1%
16.28%
27.91%
12.4%
20.16%
18.6%
10.08%
13.95%
End
20.16%
15.5%
13.95%
hospitalization
75.97%
15.5%
17.83%
6.98%

(c)
