# Supplementary material for: Care Pathways After Acute Myocardial Infarction: A Gender-Based Perspective
Source: J Clin Med. 2026 Mar 28;15(7):2592. doi: 10.3390/jcm15072592 (PMC13073914; doi:10.3390/jcm15072592)
Supplement: Supplementary file 1 [file jcm-15-02592-s001.zip › Table S1.pdf]

Table S1. Baseline sociodemographic and clinical characteristics of patients with a first AMI, overall and stratified by gender.

N, %	Overall		Women		Men		p values
Population	4298	100.00	1213	28.22	3085	71.78	<0.001
Age at the event (mean, sd)*	70.52	13.30	76.46	12.50	68.20	12.86	<0.001
Nacionality							
Spanish	4125	96.00	1185	97.69	2940	95.30	<0.001
Immigrant	172	4.00	28	2.31	144	4.67	
Socioeconomic status							
Pensioners < 18,000€ per year	2195	51.07	846	69.74	1349	43.73	<0.001
Pensioners > 18,000€ per year	880	20.47	176	14.51	704	22.82	
Unemployed	194	4.51	46	3.79	148	4.80	
Actives < 18,000€ per year	438	10.19	60	4.95	378	12.25	
Actives >18,000€ per year	445	10.35	42	3.46	403	13.06	
Other socioeconomic level	146	3.40	43	3.54	103	3.34	
Residential area							
Urban	3020	70.27	882	72.71	2138	69.30	0.028
Rural	1278	29.73	331	27.29	947	30.70	
Institutionalised	285	6.63	144	11.87	141	4.57	<0.001
Comorbidities							
Hypertension	3013	70.10	964	79.47	2049	66.42	<0.001
Dyslipemia	4195	97.60	1160	95.63	3035	98.38	<0.001
Diabetes Mellitus	2142	49.84	588	48.47	1554	50.37	0.263
Heart failure	536	12.49	227	18.71	309	10.02	<0.001
Chronic Obstructive Pulmonary Disease	439	10.23	77	6.35	362	11.73	<0.001
Depression	670	15.62	311	25.64	359	11.64	<0.001
Chronic Kidney Disease	1015	23.66	330	27.21	685	22.20	<0.001
Cirrhosis	152	3.54	45	3.71	107	3.47	0.691
Osteoporosis	342	7.97	304	25.06	38	1.23	<0.001
Dementia	154	3.59	88	7.25	66	2.14	<0.001
Num Pathologies (mean, sd)*	6.54	2.93	7.56	2.96	6.15	2.82	<0.001
Complexity							
Level 1 (minimun)	259	6.04	49	4.04	210	6.81	<0.001
2	1203	28.04	371	30.59	832	26.97	
3	1019	23.75	313	25.80	706	22.88	
4	1076	25.08	299	24.65	777	25.19	
Level 5 (maximun)	733	17.09	177	14.59	556	18.02	
Morbidity burden (mean, sd)*	12.38	6.25	14.15	6.3	11.68	6.09	<0.001
Weight (mean, sd)*	78.06	16.44	68.34	14.95	81.81	15.42	<0.001
Height (mean, sd)*	163.43	15.82	153.12	13.77	167.35	14.76	<0.001
Body Mass Index: Missing values 1408							
Underweight	18	0.62	10	0.82	8	0.26	<0.001
Normal range	558	19.31	195	16.08	363	11.77	
Overweight	1287	44.53	300	24.73	987	31.99	

Obese	1027	35.54	287	23.66	740	23.99	
Smoking habit: Missing values 878	684	20.00	130	10.72	554	17.96	<0.001
Pharmacological treatment							
Beta-Blockers (C07)	3528	82.08	953	78.57	2575	83.47	<0.001
Lipid-lowering (C10)	3972	92.42	1058	87.22	2914	94.46	<0.001
Anti-platelet agents (B01AC)	4064	94.56	1105	91.10	2959	95.92	<0.001
ACE-I/ARBs (C09)	3301	76.80	908	74.86	2393	77.57	0.058
Antidiabetics (A10)	3961	92.16	1110	91.51	2851	92.41	0.32
Diuretics (C03)	1815	42.23	681	56.14	1134	36.76	<0.001
Pharmacological burden*	11.10	3.93	12.31	3.99	10.62	3.80	<0.001

AMI: Acute myocardial infarction. N: number %: percentage. *Continuous variables expressed as mean, standard deviation (sd). p: statistical significance $p<0.05$. Pearson's Chi-squared test. Student's T-test
