# Supplementary material for: Care Pathways After Acute Myocardial Infarction: A Gender-Based Perspective
Source: J Clin Med. 2026 Mar 28;15(7):2592. doi: 10.3390/jcm15072592 (PMC13073914; doi:10.3390/jcm15072592)
Supplement: Supplementary file 1 [file jcm-15-02592-s001.zip › Table S2..pdf]

Table S2. Multivariable logistic regression analysis of factors associated with the type of first contact after AMI (scheduled visit versus emergency department), stratified by sex.

	Men			Women		
**Characteristic**	**OR**	**95% CI**	**p-value**	**OR**	**95% CI**	**p-value**
Age	1.01	1.00, 1.01	**0.055**	1.02	1.00, 1.03	**0.005**
Socioeconomic status						
≥ 18000 € per year	0.90	0.77, 1.06	0.2	0.90	0.65, 1.24	0.5
(Ref. < 18000 € per year)						
Residential area						
Urban	0.59	0.49, 0.70	**<0.001**	0.68	0.50, 0.92	**0.014**
(Ref. Rural)						
Comorbidities						
Hypertension						
(Ref. No hypertensive)	1.06	0.89, 1.26	0.5	0.82	0.58, 1.14	0.2
Dyslipemia						
(Ref. No dyslipidemia)	0.94	0.48, 1.76	0.9	1.36	0.70, 2.55	0.3
Diabetes Mellitus						
(Ref. No diabetes)	0.94	0.80, 1.10	0.4	0.99	0.76, 1.28	>0.9
Overweight						
(Ref. No overweigth)	0.92	0.74, 1.15	0.5	0.82	0.59, 1.15	0.2
Ischemic heart disease						
(Ref. No ischemic heart disease)	0.78	0.60, 1.01	0.066	0.69	0.46, 1.02	0.068
Chronic Obstructive Pulmonary Disease						
(Ref. No COPD)	0.93	0.73, 1.19	0.6	1.03	0.62, 1.78	>0.9
Depression						
(Ref. No depression)	1.38	1.08, 1.78	**0.011**	1.07	0.80, 1.44	0.7
Chronic Kidney Disease						
(Ref. No Chronic Kidney Disease)	0.82	0.67, 0.99	**0.041**	1.10	0.81, 1.51	0.5
Cirrhosis						
(Ref. No cirrhosis)	0.74	0.50, 1.11	0.14	1.43	0.73, 3.04	0.3
Dementia						
(Ref. No dementia)	0.65	0.39, 1.12	0.11	1.41	0.82, 2.53	0.2

Abbreviations: Ref = reference category, CI = Confidence Interval, OR = Odds Ratio
