# Supplementary material for: Care Pathways After Acute Myocardial Infarction: A Gender-Based Perspective
Source: J Clin Med. 2026 Mar 28;15(7):2592. doi: 10.3390/jcm15072592 (PMC13073914; doi:10.3390/jcm15072592)
Supplement: Supplementary file 1 [file jcm-15-02592-s001.zip › Table S3.pdf]

**Table S3: First point of contact in post-discharge care after AMI before and during the COVID-19 pandemic.**

| N, % | *Before COVID* | | *During COVID* | |
|---|---|---|---|---|
| Emergency | 701 | 31.86 | 654 | 29.67 |
| General practitioner | 567 | 25.77 | 618 | 28.04 |
| Specialist | 534 | 24.27 | 503 | 22.82 |
| Primary Care nurse | 367 | 16.68 | 403 | 18.28 |
| Hospitalization | 31 | 1.41 | 26 | 1.18 |

AMI: acute myocardial infarction. N: number %: percentage. p: statistical significance $p<0.05$. Pearson's Chi-squared test.
