# Supplementary material for: Care Pathways After Acute Myocardial Infarction: A Gender-Based Perspective
Source: J Clin Med. 2026 Mar 28;15(7):2592. doi: 10.3390/jcm15072592 (PMC13073914; doi:10.3390/jcm15072592)
Supplement: Supplementary file 1 [file jcm-15-02592-s001.zip › Table S4.pdf]

Table S4. Baseline sociodemographic and clinical characteristics of patients experiencing clinical outcomes after acute myocardial infarction, overall and stratified by gender.

N, %	Overall		Women		Men		p values
**Population**	352	8.19	129	10.63	223	7.23	**<0.001**
**Age at the event (mean, sd)***	78.15	12.85	83.34	10.27	75.15	13.25	**<0.001**
**Nationality**							
Native	346	98.30	127	98.45	219	98.21	0.865
Immigrant	6	1.70	2	1.55	4	1.79	
**Socioeconomic status**							
Pensioners < 18,000€ per year	240	68.18	107	82.95	133	59.64	**<0.001**
Pensioners > 18,000€ per year	69	19.60	14	10.85	55	24.66	
Unemployed	9	2.56	5	3.88	4	1.79	
Actives < 18,000€ per year	14	3.98	0	0.00	14	6.28	
Actives >18,000€ per year	16	4.55	2	1.55	14	6.28	
Other socioeconomic level	4	1.14	1	0.78	3	1.35	
**Residential area**							
Urban	249	70.74	97	75.19	152	68.16	0.162
Rural	103	29.26	32	24.81	71	31.84	
**Institutionalised**	52	14.77	28	21.71	24	10.76	**0.005**
**Comorbidities**							
Hypertension	262	74.43	103	79.84	159	71.30	0.077
Dyslipemia	321	91.19	112	86.82	209	93.72	**0.028**
Diabetes Mellitus	201	57.10	66	51.16	135	60.54	0.087
Heart failure	89	25.28	44	34.11	45	20.18	**0.003**
Chronic Obstructive Pulmonary Disease	45	12.78	12	9.30	33	14.80	0.145
Depression	58	16.48	30	23.26	28	12.56	**0.008**
Chronic Kidney Disease	123	34.94	47	36.43	76	34.08	0.606
Cirrhosis	8	2.27	5	3.88	3	1.35	0.121
Osteoporosis	44	12.50	40	31.01	4	1.79	**<0.001**
Dementia	29	8.24	14	10.85	15	6.73	0.166
**Num Pathologies (mean, sd)***	7.29	3.27	8.13	3.34	6.82	3.17	**<0.001**
**Complexity**							
Level 1 (minimun)	18	5.11	5	3.88	13	5.83	0.513
Level 5 (maximun)	56	15.91	19	14.73	37	16.59	
**Morbidity burden (mean, sd)***	14.80	7.43	16.26	7.30	13.97	7.38	**0.006**
**Weight (mean, sd)***	73.77	16.73	64.92	15.86	78.68	15.14	**<0.001**
**Height (mean, sd)***	160.51	15.98	150.26	19.63	166.04	10.00	**<0.001**
**IMC:** Missing values 142							
Underweight	3	0.85	2	1.55	1	0.45	0.483
Normal range	37	10.51	10	7.75	27	12.11	
Overweight	99	28.13	36	27.91	63	28.25	
Obese	71	20.17	24	18.60	47	21.08	
**Smoking habit**: Missing values 92	27	7.67	6	4.65	21	9.42	0.141

N: number %: percentage. *Continuous variables expressed as mean, standard deviation (sd).
p: statistical significance $p<0.05$. Pearson's Chi-squared test. Student's T-test.
