# Supplementary material for: Care Pathways After Acute Myocardial Infarction: A Gender-Based Perspective
Source: J Clin Med. 2026 Mar 28;15(7):2592. doi: 10.3390/jcm15072592 (PMC13073914; doi:10.3390/jcm15072592)
Supplement: Supplementary file 1 [file jcm-15-02592-s001.zip › Table S5.pdf]

Table S5. Incidence of clinical outcomes within 90 days after acute myocardial infarction, stratified by gender.

N (%)	Overall<br>N=4,298	Men<br>N=3,085	Women<br>N=1,213	p-values
**Death within 90 days**	146 (3.4%)	88 (2.85% of men)	58 (4.78% of women)	**0.0023**
**Death due to AMI within 90 days**	82 (1.9%)	52 (1.69% of men)	30 (2.47% of women)	0.1153
**Recurrent AMI from day 7 to 90**	243 (5.7%)	158 (5.12% of men)	85 (7.01% of women)	**0.0195**

AMI: Acute myocardial infarction. N: number %: percentage. p: statistical significance $p<0.05$. Pearson's Chi-squared test.
