# Supplementary material for: Care Pathways After Acute Myocardial Infarction: A Gender-Based Perspective
Source: J Clin Med. 2026 Mar 28;15(7):2592. doi: 10.3390/jcm15072592 (PMC13073914; doi:10.3390/jcm15072592)
Supplement: Supplementary file 1 [file jcm-15-02592-s001.zip › Table S6.pdf]

Table S6. Average follow-up time (in days) after acute myocardial infarction, overall and stratified by gender.

**Overall population N=4295**		Women N=1212	Men N=3083	p-value
Mean (SD)	49.73 (26.00)	50.7 (26.1)	49.3 (26.0)	0.121
**Clinical Outcomes n=352**		Women N=129	Men N=223	p-value
Mean (SD)	44.28 (26.13)	42.1 (25.7)	45.5 (26.3)	0.229

N: number. SD: standard deviation. p: statistical significance $p<0.05$. Student's T-test.
