# Supplementary material for: Care Pathways After Acute Myocardial Infarction: A Gender-Based Perspective
Source: J Clin Med. 2026 Mar 28;15(7):2592. doi: 10.3390/jcm15072592 (PMC13073914; doi:10.3390/jcm15072592)
Supplement: Supplementary file 1 [file jcm-15-02592-s001.zip › Table S7.pdf]

Table S7. First point of contact in post-discharge care among patients experiencing clinical outcomes after acute myocardial infarction, stratified by gender.

	Overall		Women		Men		p values
	N	%	N	%	N	%	
General practitioner	106	30.11	41	31.78	65	29.15	0.690
Primary Care nurse	59	16.76	25	19.38	34	15.25	0.394
Specialist	81	23.01	34	26.36	47	21.08	0.316
Emergency	81	23.01	20	15.50	61	27.35	**0.016**
Hospitalisation	25	7.10	9	6.98	16	7.17	1.000

N: number %: percentage. p: statistical significance $p<0.05$. Pearson's Chi-squared test.
